# Clinical Management of Peri-Aortitis Following Endovascular Aortic Repair for Abdominal Aortic Aneurysm

**DOI:** 10.3400/avd.oa.24-00143

**Published:** 2025-05-13

**Authors:** Yuriko Takeuchi, Noriyasu Morikage, Ryunosuke Sakamoto, Takahiro Mizoguchi, Makoto Samura, Takasuke Harada, Hiroshi Kurazumi, Ryo Suzuki, Kotaro Suehiro, Kimikazu Hamano

**Affiliations:** 1 Division of Vascular Surgery, Department of Surgery and Clinical Science, Yamaguchi University Graduate School of Medicine, Ube, Yamaguchi, Japan; 2 Department of Cardiovascular Surgery, Kansai Medical University, Hirakata, Osaka, Japan

**Keywords:** peri-aortitis, endovascular aneurysm repair, inflammatory abdominal aortic aneurysm, steroid, infection

## Abstract

**Objectives:** Peri-aortitis following endovascular aneurysm repair (EVAR) is a rare phenomenon with unclear pathogenesis. In this study, we investigated its clinical features and sac prognosis.

**Methods:** A retrospective analysis was conducted on 1369 EVAR. Peri-aortitis was defined using post-EVAR computed tomography. Clinical and imaging data were assessed.

**Results:** Peri-aortitis following EVAR was identified in 12 patients (0.89%) with a mean age of 74 ± 8.9 years; 83.3% were male, and 41.7% had allergic or autoimmune histories. There were eight symptomatic cases (66.7%), including seven with fever, three with back or abdominal pain, and one with hydronephrosis. Precautionary antibiotic treatment was administered in five febrile cases. Although persistent and recurrent inflammation was observed in two cases (16.7%) each, inflammation resolved spontaneously in seven patients (58.3%). One (8.3%) needed steroid therapy for severe back pain. Aneurysm shrinkage was observed in seven cases (58.3%), while enlargement was noted in one case (8.3%) with type II endoleak. No correlation was found between aneurysm growth and peri-aortitis development.

**Conclusions:** Peri-aortitis following EVAR may present significant challenges, including differentiation from infection, management of symptomatic cases requiring medical therapy, and addressing recurrences. Accurate diagnosis, individualized treatment, and meticulous follow-up are essential for favorable outcomes.

## Introduction

De novo development of peri-aortitis following endovascular aneurysm repair (EVAR) is a rarely described phenomenon and its pathogenesis remains unclear.^1)^ Endovascular grafts elicit a foreign body reaction, which may contribute to the development of de novo peri-aortitis due to this additional stimulus. In animals, endovascular repair triggers an immediate local inflammatory response around the stent graft, within the aneurysm and perianeurysmal tissue.^2,3)^ Here, we analyze 12 cases observed at our institution, emphasizing their clinical characteristics and sac prognosis.

## Materials and Methods

**Figure 1:**
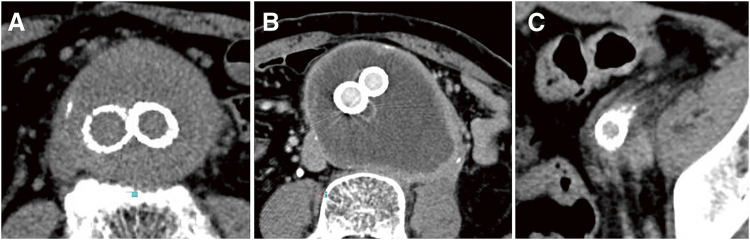
Fig. 1 CT images of the peri-aortitis after EVAR. (**A**) Soft tissue mass surrounding the aorta. (**B**) Mild aortic wall thickening with contrast enhancement. (**C**) Feathery appearance around the aorta. EVAR: endovascular aneurysm repair

We retrospectively analyzed the clinical features and follow-up computed tomography (CT) findings of patients with peri-aortitis following EVAR among 1369 EVAR procedures performed at our institution between April 2007 and March 2023. The definition of peri-aortitis after EVAR was based on previous reports and included cases exhibiting any of the following CT findings (**Fig. 1**): a soft tissue mass surrounding the aorta, mild wall thickening with contrast enhancement, or a feathery appearance around the aorta.^1)^ The timing of onset following EVAR was not restricted. Changes in inflammation during the follow-up period were evaluated based on alterations in the CT findings. This study was approved by our institutional review board (H2019-062-3).

## Results

Peri-aortitis following EVAR was documented in 12 (0.89%) of the 1369 patients. **Table 1** shows the clinical presentation of each patient with peri-aortitis after EVAR. The mean age of the patients was 74 ± 8.9 years, with 10 (83.3%) being male and 5 (41.7%) having a history of allergic or autoimmune diseases. The main body of stent grafts used were the Excluder (W.L. Gore & Associates, Flagstaff, AZ, USA) and the Endurant (Medtronic, Minneapolis, MN, USA), each used in six cases. Symptomatic cases accounted for eight (66.7%) of the total cases, including seven cases with fever, three with back or abdominal pain, and one with hydronephrosis. At onset, the mean white blood cell (WBC) count was 10462 ± 5340/μL, and the mean C-reactive protein (CRP) level was 13.7 ± 10.6 mg/dL. Serum IgG4 levels were ≥135 mg/dL in three cases (27.2%), and antinuclear antibodies were positive in six cases (54.5%). CT imaging at onset revealed a soft tissue mass surrounding the aorta in four cases (33.3%), mild wall thickening with contrast enhancement in five cases (41.7%), and a feathery appearance around the aorta in three cases (25.0%). The mean preoperative sac diameter was 60.8 ± 16.6 mm. The mean duration to onset following EVAR was 3.9 ± 4.9 months.

**Table:** Table 1 Clinical presentation of each case of peri-aortitis following endovascular aneurysm repair

Case	Age	Sex	Allergy/ autoimmune diseases	Stent graft	Symptoms	WBC, /μL	CRP, mg/dL	Serum IgG4, mg/dL	Antinuclear antibodies	Antibiotic therapy
1	68	Male	Food allergy	Excluder	Hydronephrosis (at recurrence: fever, back pain)	8130	0.77	24.7	+	No
2	53	Male	Rheumatic disease	Excluder	Fever	12600	18.10	NA	+	Yes
3	67	Male	Drug and food allergy	Endurant	Fever	22580	19.94	154.0	+	No
4	80	Female	Food allergy	Excluder	Fever, abdominal pain	10500	28.02	79.0	+	Yes
5	64	Male	None	Excluder	Fever, abdominal pain	18870	31.70	52.8	–	Yes
6	72	Male	None	Endurant	Fever	6740	16.68	89.3	+	Yes
7	78	Female	None	Endurant	None	6090	0.33	59.4	–	No
8	82	Male	None	Endurant	Fever	6050	9.14	115.0	–	No
9	81	Male	None	Endurant	Fever	8010	13.33	46.8	–	Yes
10	79	Male	None	Endurant	None	12060	20.23	71.8	NA	No
11	78	Male	Allergy to iodinated contrast medium	Excluder	None (at recurrence: none)	7440	0.56	513.0	–	No
12	80	Male	None	Excluder	None	6470	5.48	376.0	+	No

WBC: white blood cell; CRP: C-reactive protein; NA: not attenuated

**Figure 2:**
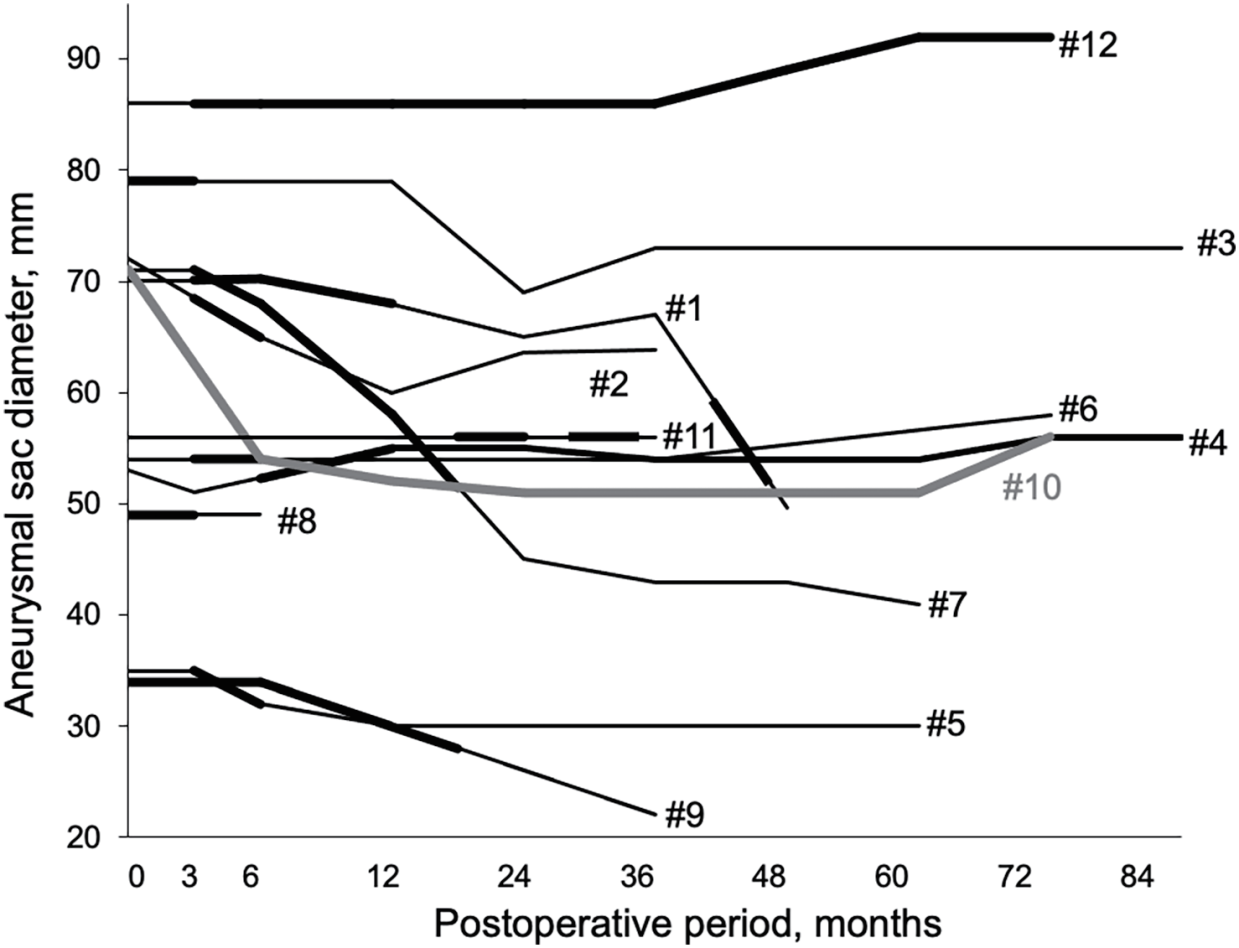
Fig. 2 Absence of correlation between aneurysm growth and development of peri-aortitis. The bold line indicates the period during which inflammation was observed on CT imaging. The case numbers (#) match those in **Table 1**

**Figure 2** shows the correlation between the postoperative transition in aneurysm size and the development of peri-aortitis. The mean follow-up period was 73.2 ± 29.6 months. Inflammation resolved throughout the follow-up period in seven patients (58.3%), with a mean duration of 6.3 ± 3.8 months before resolution. One patient (case #4) showed improvement without complete resolution of inflammation, two patients (#10 and #12) had persistent inflammation without change, and two patients (#1 and #11) experienced recurrence of inflammation after initial resolution. In one of the two recurrent cases (#1), oral steroid therapy was administered to treat back pain caused by inflammation rather than hydronephrosis during the peri-aortitis recurrence. Type II endoleaks were observed in three cases (#2, #4, and #8), whereas type Ia endoleaks occurred in two cases (#3 and #10). Case #3 was successfully treated with an endovascular procedure, whereas case #10 died of rupture. Case #12 was the only instance in which the sac diameter increased by more than 5 mm compared with its preoperative size, and this enlargement was related to a type II endoleak. Sac shrinkage >5 mm was observed in seven patients (58.3%). As shown in **Fig. 2**, no correlation was observed between aneurysm growth and the development of peri-aortitis.

## Discussion

Peri-aortitis following EVAR is a rare complication.^1)^ Potential causes include the stent graft material, intimal injury during device deployment, and intraluminal thrombus involvement.^1,4)^ This condition typically presents with elevated CRP levels and inflammation-related symptoms, including abdominal or back pain. Additionally, inflammation may extend to the ureters or inferior vena cava, causing hydronephrosis or lower limb edema.^5–9)^ Pharmacological treatments are indicated for symptomatic cases.^5–11)^ Steroids and tamoxifen have been reported as pharmacological treatments, with randomized controlled trials (RCTs) demonstrating the superiority of steroids over tamoxifen in idiopathic retroperitoneal fibrosis.^12)^ In the present cases, one patient required oral steroid therapy to manage back pain during peri-aortitis recurrence.^13)^ However, the use of steroids for abdominal aortic aneurysms has been reported to increase the risk of rupture and requires careful monitoring during treatment.^14)^

Reports have described cases in which open surgery was performed for peri-aortic soft tissue mass observed after EVAR, leading to a diagnosis of xanthogranuloma, or where occult IgG4-related inflammatory abdominal aortic aneurysm (IAAA) became apparent after a certain period following EVAR.^15,16)^ These findings suggest that peri-aortitis following EVAR may encompass a wide spectrum of pathologies. In this study, while no patients were definitively diagnosed with IgG4-related IAAA, 27.2% showed elevated serum IgG4 levels, indicating a possible relationship between IgG4-related IAAA and peri-aortitis following EVAR.

Stent graft infection is a key differential diagnosis. Distinguishing between graft infection and peri-aortitis using CT can be challenging. Graft infection is typically characterized by ectopic gas, peri-graft inflammation and fluid, thickening of the adjacent bowel, and pseudoaneurysm formation, whereas peri-aortitis generally presents with aortic wall thickening and a low-density, mildly enhancing soft tissue mass surrounding the aorta.^17,18)^ Fluorodeoxyglucose positron emission tomography-CT can detect fluorodeoxyglucose uptake during inflammation and infection, making complete differentiation challenging. However, labeled white blood cell imaging has been reported to show specific uptake in infections, and is considered a useful diagnostic tool.^10,19)^ In the present cases, blood cultures and procalcitonin levels were measured in seven patients with fever to differentiate infection. Although the results were negative in these cases, antibiotics were administered in five cases due to the potential severity of stent graft infections, despite antibiotic therapy not being clinically necessary.

In this study, no correlation was observed between aneurysm diameter progression and the degree of inflammation, which is consistent with previous reports.^1)^ However, in a previous study, 18% of the patients with post-EVAR sac enlargement who underwent open conversion after EVAR met the diagnostic criteria for IgG4-related vascular diseases.^20)^ Given that inflammatory aortic aneurysms and peri-aortitis following EVAR share a common trigger of inflammation, the possibility that peri-aortitis following EVAR contributes to sac enlargement cannot be ruled out. Post-implantation syndrome (PIS) is characterized by an early systemic inflammatory response triggered by EVAR. A recent review reported that PIS was associated with higher 30-day mortality and an increased incidence of major adverse cardiovascular events. PIS is often defined as a condition occurring shortly after EVAR, presenting with a body temperature >38°C and WBC >12000/μL in the absence of signs of infection.^21)^ In the present study, all four patients with peri-aortic inflammation observed on immediate postoperative CT imaging met these criteria. Further investigation is needed to clarify whether peri-aortitis following EVAR shares a clinical trajectory with PIS. Peri-aortitis following EVAR, inflammatory abdominal aortic aneurysms, and PIS share common inflammatory features, but their pathophysiological mechanisms remain incompletely understood. At present, they are considered distinct conditions that should be differentiated.

Among the 1369 EVAR procedures included in this study, all cases of peri-aortitis following EVAR occurred before 2017. Since 2018, our institution has implemented a preemptive embolization strategy for all EVAR cases involving patent aneurysmal branch vessels to reduce the risk of sac enlargement caused by type II endoleaks. Notably, no cases of peri-aortitis following EVAR have been reported in patients who underwent this preventive approach. Although the microcirculatory flow from branch vessels and the resulting changes in the intraluminal thrombus within the aneurysmal sac may contribute to the development of peri-aortitis, definitive conclusions cannot yet be drawn.

## Conclusion

Based on our experience, peri-aortitis following EVAR often resolves spontaneously and shows no correlation with changes in aneurysm diameter. Although it is generally considered a benign condition, differentiating it from infection remains a critical challenge. Furthermore, some cases have symptomatic recurrence or require pharmacological intervention. Therefore, meticulous evaluation, close monitoring, and appropriate management are necessary.

## Declarations

### Disclosure statement

The authors have no competing interests.

### Author contributions

Study conception: KH, NM, MS, YT

Data collection: KS, RSu, HK, TH, MS, TM, RSa, YT

Analysis: KS, RSu, HK, TH, MS, TM, RSa, YT

Investigation: KS, RSu, HK, TH, MS, TM, RSa, YT

Manuscript preparation: MS, YT

Funding acquisition: KH, NM

Critical review and revision: all authors

Final approval of the article: all authors

Accountability for all aspects of the work: all authors.
